# Supplementary material for: Auxin Metabolite Profiling in Isolated and Intact Plant Nuclei
Source: Int J Mol Sci. 2021 Nov 16;22(22):12369. doi: 10.3390/ijms222212369 (PMC8620152; doi:10.3390/ijms222212369)
Supplement: Supplementary file 1 [file ijms-22-12369-s001.zip › ijms-1434205-supplementary 2.pdf]

International Journal of *Molecular Sciences*

MDPI

Supplementary Material

Auxin Metabolite Profiling in Isolated and Intact Plant Nuclei

Vladimír Skalický [1], Tereza Vojtková [1], Aleš Pěnčík [1], Jan Vrána [2], Katarzyna Juzoń [3], Veronika Koláčková [2], Michaela Sedlářová [4], Martin F. Kubeš [1,5,*,†] and Ondřej Novák [1,*]

1 Laboratory of Growth Regulators, Institute of Experimental Botany of the Czech Academy of Sciences and Faculty of Science, Palacký University, Šlechtitelů 27, CZ-78371 Olomouc, Czech Republic; vladimir.skalicky@upol.cz (V.S.); vojtkova@ueb.cas.cz (T.V.); ales.pencik@upol.cz (A.P.)
2 Institute of Experimental Botany of the Czech Academy of Sciences, Centre of the Region Haná for Biotechnological and Agricultural Research, Šlechtitelů 31, 77900 Olomouc, Czech Republic; jan.vrana@fno.cz (J.V.); kolackova@ueb.cas.cz (V.K.)
3 Department of Biotechnology, The Franciszek Górski Institute of Plant Physiology, Polish Academy of Sciences, Niezapominajek 21, 30-239 Krakow, Poland; k.juzon@ifr-pan.edu.pl
4 Department of Botany, Faculty of Science, Palacký University, Šlechtitelů 27, 78371 Olomouc, Czech Republic; michaela.sedlarova@upol.cz (M.S.)
5 School of Life Sciences, University of Warwick, Coventry CV4 7AL, UK
* Correspondence: martin.kubes@uhk.cz (M.F.K.); novako@ueb.cas.cz (O.N.); Tel.: +420-585-634-852 (O.N.)
† Current Address: Department of Biology, Faculty of Science, University of Hradec Králové, Rokitanského 62, 50003 Hradec Králové, Czech Republic.

Citation: Skalický, V.; Vojtková, T.; Pěnčík, A.; Vrána, J.; Juzoń, K.; Koláčková, V.; Sedlářová, M.; Kubeš, M.F.; Novák, O. Auxin Metabolite Profiling in Isolated and Intact Plant Nuclei. *Int. J. Mol. Sci.* **2021**, *22*, 12369. https://doi.org/10.3390/ijms222212369

Academic Editor: Massimo Maffei

Received: 8 October 2021
Accepted: 12 November 2021
Published: 16 November 2021

Publisher's Note: MDPI stays neutral with regard to jurisdictional claims in published maps and institutional affiliations.

Copyright: © 2021 by the authors. Licensee MDPI, Basel, Switzerland. This article is an open access article distributed under the terms and conditions of the Creative Commons Attribution (CC BY) license (http://creativecommons.org/licenses/by/4.0/).

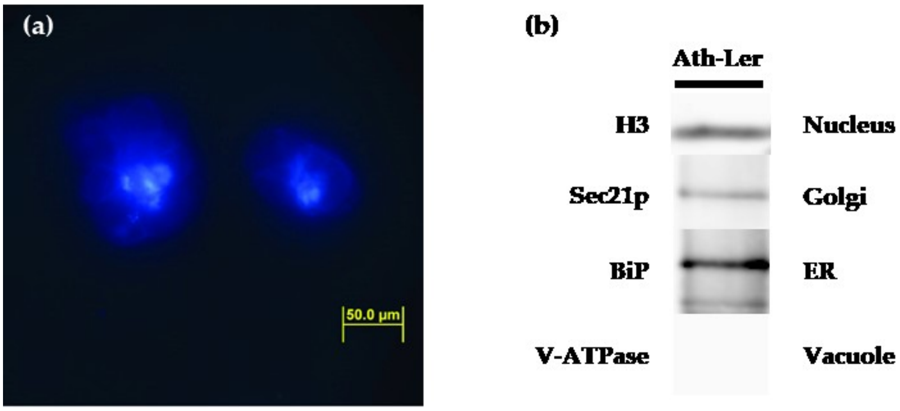

Figure S1. Isolation of nuclei according to the Method S1 [1] and purity control of isolated nuclei from *Arabidopsis thaliana* ecotype Landsberg *erecta* (*Ath*-L*er*) cell line by immunoblotting. (**a**) *Ath*-L*er* cells after lysis step with not properly released cell nuclei (stained with DAPI), (**b**) nuclear fraction was immunoblotted with anti-Histone 3 (H3) antibody to confirm enrichment of nuclei and following antibodies were used to disprove presence of contaminating organelles in particular fraction: Anti-Coatomer subunit gamma (Sec21p) for presence of Golgi complex, anti-Lumenal-binding protein (BiP) for presence of endoplasmic reticulum (ER) and finally anti-Epsilon subunit of tonoplast H+ATPase (V-ATPase) for presence of vacuole.

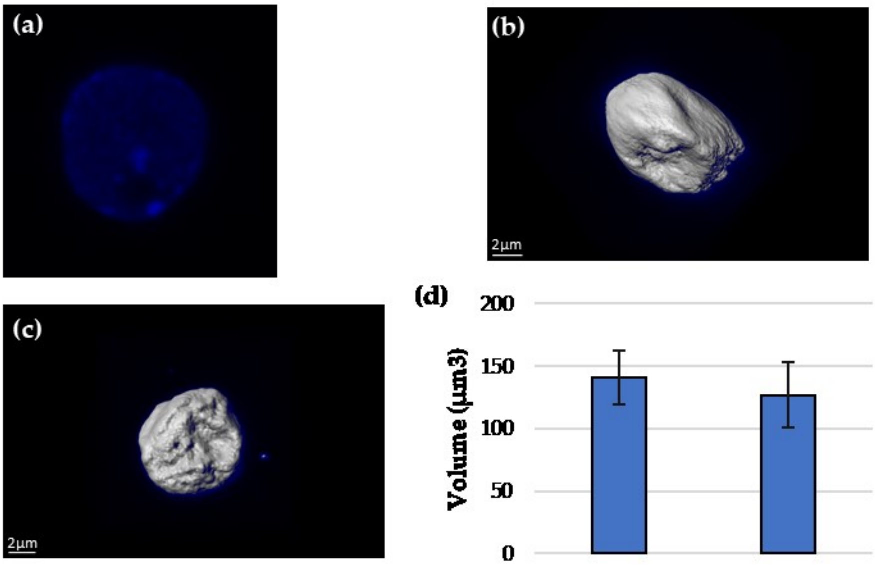

Figure S2. 3D reconstruction of nuclei from *Ath*-L*er* cell line isolated by flow cytometry method. (**a**) Individual optical section of an intact nuclei, (**b**,**c**) representative 3D reconstructions of nuclei, (**d**) average values of calculated nuclear volumes (two independent isolations of nuclei). Volume analysis was done based on 3D reconstructed nuclei(n=200). Error bars indicate SD (n=3).

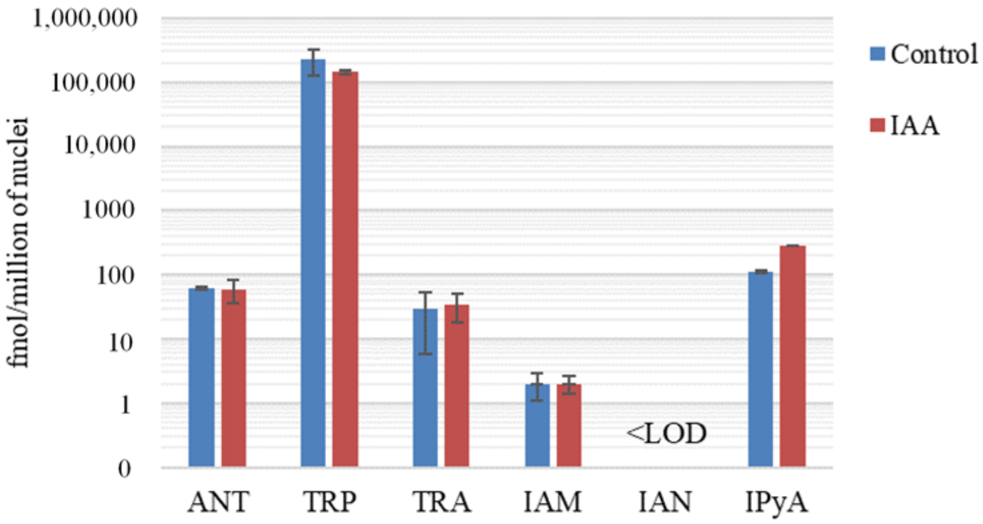

Figure S3. Endogenous levels (fmol/million of nuclei) of auxin precursors in nuclei isolated from *Ath*-L*er* protoplasts untreated (Control) and treated (IAA) with indole-3-acetic acid (n=3). Error bars indicate SD.

Table S1. Endogenous levels (fmol/million of nuclei) of auxin metabolites and their relative distribution in nuclear fractions isolated from *Ath*-L*er* and BY-2 cells.

Compound	***Ath*-L*er* cell line**		**BY-2 cell line**	
	Auxin levels [1] (fmol/1x10^6 nuclei)	Rel. Distribution (%)	Auxin levels [1] (fmol/1x10^6 nuclei)	Rel. Distribution (%)
ANT	61.09 ± 2.07	0.027%	58.22 ± 0.82	0.0085%
TRP	225,309.76 ± 100,381.48	99.6%	685,351.32 ± 185,482.78	99.8%
TRA	29.83 ± 23.99	0.013%	100.58 ± 9.59	0.015%
IAM	1.97 ± 0.89	0.0009%	n.d.	n.d.
IAN	n.d.	n.d.	n.d.	n.d.
IPyA	111.52 ± 3.69	0.049%	829.82 ± 190.86	0.12%
IAA	397.87 ± 124.09	0.18%	27.31 ± 13.69	0.0040%
oxIAA	20.15 ± 4.01	0.0089%	6.27 ± 3.06	0.0009%
IAAsp	362.24 ± 27.55	0.16%	8.80 ± 1.60	0.0013%
IAGlu	19.38 ± 7.04	0.0086%	2.12 ± 0.14	0.0003%
IAA-glc	n.d.	n.d.	n.d.	n.d.
oxIAA-glc	n.d.	n.d.	n.d.	n.d.

[1] Values are means ± SD (n=4); n.d. – not detected.

Table S2. Endogenous levels (pmol/g of fresh weight) of auxin metabolites in *Ath*-L*er* cells.

**Compound**	***Ath*-L*er* cell line Auxin levels [1] (pmol/g)**
ANT	n.d.
TRP	18,233.64 ± 1227.86
TRA	0.61 ± 0.21
IAM	0.26 ± 0.04
IAN	3.30 ± 0.38
IPyA	102.22 ± 19.54
IAA	1.35 ± 0.66
oxIAA	7.60 ± 4.55
IAAsp	2.35 ± 0.41
IAGlu	n.d.
IAA-glc	n.d.
oxIAA-glc	n.d.

[1] Values are means ± SD (n=5); n.d. – not detected.

Method S1. Alternative methods of cell nuclei isolation

S1.1. Alternative method of nuclei isolation using differential centrifugation

Samples of cell nuclei were prepared according to Xu and Copeland [1] with minor modifications. Briefly, cells were filtered and resuspended in cold lysis buffer (20 mM Tris-HCl pH 7.4, 25 % (v/v) glycerol, 20 mM KCl, 2 mM EDTA pH 7.5, 2.5 mM $MgCl_2$, and 250 mM sucrose). 1 mM 1,4-dithiothreitol (DTT) and 2 mM phenylmethylsulfonyl fluoride (PMSF) were also added before experiment (alternatively, pH was adjusted to 5.3 as described in [2]). The cell suspension was homogenized by a Dounce homogenizer and then filtered through the pre-wetted 3 layers of Miracloth membrane (20-25 μm) and pelleted by centrifugation (1500× *g*, 10 min, 4 °C). The nuclear pellet was gently resuspended in nuclear resuspension buffer (20 mM Tris-HCl pH 7.4, 25 % (v/v) glycerol, 2.5 mM $MgCl_2$ and 0.2 % (v/v) Triton X-100) and the centrifuged (1500× *g*, 10 min, 4 °C). The washing step was repeated twice. Finally, the pellet was gently resuspended in the same buffer without Triton X-100 (all components from Merck Life Science, Darmstadt, Germany). The isolated nuclei were immediately used for fluorescent microscopy and/or frozen in liquid nitrogen.

S1.2. Alternative method of nuclei isolation by flow cytometry

Samples of cell nuclei for sorting experiment were also prepared as described in Petrovská *et al.* [3] with minor modifications. Briefly, cells were fixed in 2 %(v/v) formaldehyde, gently mixed, incubated for 10 min on ice, and then pelleted by centrifugation (500× *g*, 10 min, 4 °C). Cells were grinded in mortar with pestle in lysis buffer (15 mM Tris, 2 mM Na_2EDTA, 0.5 mM EGTA, 80 mM KCl, 20 mM NaCl, 0.1 % (v/v) Triton X-100, 0.2 mM spermine and 0.5 mM spermidine pH 7.5; 14 mM 2-mercaptoethanol and 0.1 mM PMSF were added before experiment) in final ratio of 1:3. The released nuclei were filtered through 20 μm nylon mesh and stained with DAPI (4′,6-Diamidino-2-phenylindole dihydrochloride; final concentration 2 μg/ml; all components from Merck Life Science, Darmstadt, Germany). Sorting of nuclei was performed in 0.7 % (m/v) NaCl as a sheath fluid solution using BD FACS Aria II SORP flow cytometer (BD Bioscience, NY, USA). Flow cytometer was equipped with 70 μm nozzle tip, UV laser (355 nm, 100 mW) and blue laser (488 nm, 100 mW), 450/30 nm and 480/10 nm bandpass filter for DAPI and flow cytometry standard (FCS), respectively. Fluidic system was pressurized to 482.6 kPa. The population of nuclei was selected according to following optical parameters: forward and side scatter in combination with DAPI specific fluorescence. Nuclear fractions were immediately used for fluorescent microscopy and/or frozen in liquid nitrogen.

Method S2. Volume analysis of isolated cell nuclei by 3D reconstruction method

Volume analysis of sorted nuclei together with image processing and 3D-FISH were done accordingly to Koláčková *et al.* [4] and Perničková *et al.* [5]. About 50,000 nuclei in G1, G2 or S phase of the cell cycle per sample were identified and sorted using a FASCAria II SORP flow cytometer (BD Biosciences). Total genomic DNA was labelled with Texas Red using Nick Translation Kit (Roche Applied Science) according to manufacturer's instructions and applied as a probe. The probe was detected with anti-digoxigenin-fluorescein (Roche Applied Science).

Selected nuclei were optically sectioned using an inverted motorized microscope Olympus IX81 equipped with a Fluoview FV1000 confocal system (Olympus, Tokyo, Japan) and FV10-ASW software, lasers and filters were set for DAPI scanning (λex/em = 405/430-470 nm). For each nucleus, 80-120 optical sections in 160-200 nm step were taken and then merged into a 3D model. Subsequent analyses were performed using Imaris 9.2 software (Bitplane, Oxford Instruments, Zurich, Switzerland). The volume and center of the nucleus were determined from the rendering of primary intensity of DAPI nuclei staining using function 'Surfaces'. 'Display Adjustment' was used to adjust the channel contrast, and thus to improve the visualization of all analyzed objects.

References

1. Xu, F.; Copeland, C. Nuclear extraction from Arabidopsis thaliana. *BIO-PROTOCOL* **2012**, *2*, https://doi.org/10.21769/bioprotoc.306 .
2. Saxena, P. K.; Fowke, L. C.; King, J. An efficient procedure for isolation of nuclei from plant protoplasts. *Protoplasma* **1985**, *128*, 184–189, https://doi.org/10.1007/bf01276340.
3. Petrovská, B.; Jeřábková, H.; Chamrád, I.; Vrána, J.; Lenobel, R.; Uřinovská, J.; Šebela, M.; Doležel, J. Proteomic analysis of barley cell nuclei purified by flow sorting. *Cytogenet. ancyd genome Res.* **2014**, *143*, 78–86, https://doi.org/10.1159/000365311.
4. Koláčková, V.; Perničková, K.; Vrána, J.; Duchoslav, M.; Jenkins, G.; Phillips, D.; Turkosi, E.; Šamajová, O.; Sedlářová, M.; Šamaj, J.; Doležel, J.; Kopecký, D. Nuclear disposition of alien chromosome introgressions into wheat and rye using 3D-FISH. *Int. J. Mol. Sci.* **2019**, *20*, 4143, https://doi.org/10.3390/ijms20174143.
5. Perničková, K.; Koláčková, V.; Lukaszewski, A. J.; Fan, C.; Vrána, J.; Duchoslav, M.; Jenkins, G.; Phillips, D.; Šamajová, O.; Sedlářová, M.; Šamaj, J.; Doležel, J.; Kopecký, D. Instability of alien chromosome introgressions in wheat associated with improper positioning in the nucleus. *Int. J. Mol. Sci.* **2019**, *20*, 1448, https://doi.org/10.3390/ijms20061448.
